# Mental health interventions in Myanmar: a review of the academic and gray literature

**DOI:** 10.1017/gmh.2017.30

**Published:** 2018-02-19

**Authors:** A. J. Nguyen, C. Lee, M. Schojan, P. Bolton

**Affiliations:** 1 Department of Human Services, Curry School of Education, University of Virginia, Charlottesville, VA, USA; 2 Department of International Health, Johns Hopkins School of Public Health, Baltimore, MD, USA; 3 Center for Humanitarian Health, Johns Hopkins School of Public Health, Baltimore MD, USA; 4 Department of Mental Health, Johns Hopkins School of Public Health, Baltimore MD, USA

**Keywords:** Effectiveness, intervention, mental health, Myanmar, psychosocial

## Abstract

**Background.:**

Recent political changes in Myanmar provide opportunities to expand mental health (MH) services. Given Myanmar's unique situation, we felt a need to assemble and interpret available local information on MH in Myanmar to inform service design, rather than simply drawing lessons from other countries. We reviewed academic and gray literature on the experience of MH problems in Myanmar and the suitability, availability, and effectiveness of MH and psychosocial programming.

**Methods.:**

We searched: (1) Google Scholar; (2) PubMed; (3) PsychInfo; (4) English-language Myanmar journals and databases; (5) the Mental Health and Psychosocial Support (MHPSS) Network resources website; (6) websites and (7) local contacts of organizations identified during 2010 and 2013 mapping exercise of MHPSS providers; (8) the Myanmar Information Management Unit (MIMU) website; (9) University libraries in Yangon and Mandalay; and (10) identified local MH professionals.

**Results.:**

Qualitative data suggest that MH conditions resulting from stress are similar to those experienced elsewhere. Fourteen intervention evaluations were identified: three on community-level interventions, three on adult religion-based practice (meditation), four adult psychotherapeutic interventions, and four child-focused interventions. Support for the acceptability and effectiveness of interventions is mostly anecdotal. With the exception of two rigorous, randomized control trials, most evaluations had serious methodologic limitations.

**Conclusions.:**

Few evaluations of psychotherapeutic or psychosocial programs for people from Myanmar have been published in the black or gray literature. Incorporating rigorous evaluations into existing and future programs is imperative for expanding the evidence base for psychotherapeutic and psychosocial programs in this context.

## Introduction

Myanmar in South-East Asia is home to more than 50 million people. The population is diverse; while the Bamar ethnic group is nearly 70% of the total population and nearly 90% practice Buddhism, there are an additional 135 ethnic groups (Central Intelligence Agency, 2017). Formerly a British colony, the country has experienced internal conflict between the ruling military government and ethnic groups for decades (IRIN, 2012) and is now experiencing rapid social and political changes. The United Nations (UN) describes ongoing challenges to peace and security in Myanmar as ‘a complex combination of vulnerability to natural disasters, food and nutrition insecurity, armed conflict, inter-communal tensions, statelessness, displacement, trafficking and migration’. (United Nations and Partners, 2016; p. 5). The UN further highlights the exacerbating role of poverty, inequality, and discrimination in compounding vulnerabilities of affected people, as illustrated by the current humanitarian crisis in Rakhine state (OHCHR, 2017).

Given these challenges, the large burden of mental health (MH) problems in the country is not surprising. The 2015 Global Burden of Disease study reports that in Myanmar both depressive and anxiety disorders are among the top 10 contributors to years lived with disability, and both have increased over the past decade (Institute for Health Metrics and Evaluation, 2017). Most prevalence studies of MH problems have been conducted among expatriates from Myanmar. Adult refugees on the Thai border have reported significant symptoms of depression (41.8%), anxiety (40.8%), and post-traumatic stress (PTS; 4.6%; Cardozo *et al.* 2004), and 7.4% of women reported thoughts of suicide in the past month (Falb *et al.* 2013).

Currently over two million people from Myanmar live as migrants in Thailand and 103 000 refugees live in camps along the Thai border (UNHCR, n.d.). Recent political reforms have brought about greater civil society freedom, human rights improvements, and increased international engagement (Hlaing, 2012). These changes are likely to increase the number of people returning to Myanmar, while ongoing conflicts in Myanmar's ethnic areas have resulted in continued internal displacement and exposure to stressful living circumstances (Lee, 2015). Several health organizations in ethnic states provide community-based health care services in remote areas. Although many of these organizations are seeking to coordinate health service provision with the central health care system, much remains to be decided and the impacts of these decisions weigh heavily on available health and MH service resources. The 2017–2021 National Health Plan for Myanmar highlights the human resource issue, calling for an assessment of skill needs at different levels of the health system and for different cadres of workers (Ministry of Health and Sports, 2016). Given the extensive list of service needs and a need to prioritize resources, addressing MH will likely continue to be challenging. The political reforms therefore highlight both an important opportunity and an urgent need to strengthen Myanmar's health system, which has been weakened by years of underfunding (Risso-Gill *et al.* 2014).

To advance research and development in this area and to improve existing and future services, we have undertaken a rigorous review of academic and gray literature to provide relevant information on the suitability, availability, and evidence for MH and psychosocial [Mental Health and Psychosocial Support Network (MHPSS)] programming in Myanmar and to make recommendations for future programming and research. Specifically, we aimed to summarize: (1) information on the current MH service system in Myanmar; (2) perspectives of people from Myanmar on MH problems and MH services; and (3) evidence on the effectiveness of MH interventions with people from Myanmar both within and outside the country.

## Methods

We searched Google Scholar, PubMed, and PsychInfo to identify published articles and gray literature sources for information relevant to the impact or appropriateness of MH interventions in Myanmar or with people from Myanmar. We excluded studies outside Myanmar unless they focused on people from Myanmar, research covering multiple nationalities if the findings relevant to people from Myanmar could not be clearly separated, commentaries and summaries that were not related to specific programs, and studies involving only pharmacological or medical interventions.

We searched the following English-language Myanmar journals and databases for ‘mental health’ OR ‘psychosocial’: (1) *Journal of Burma Studies*; (2) *Myanmar Medical Journal*; (3) *Myanmar Journal of Medical Research*; (4) *Myanmar Health Sciences Research Journal*; and (5) BurmaLibrary.Org.

To identify relevant gray literature, we searched the MHPSS and the Myanmar Information Management Unit (MIMU) websites, as well as websites of organizations identified during a 2010 mapping exercise of MHPSS services (Burma Border Project, 2010) and a 2013 situational analysis of MHPSS in humanitarian settings in Myanmar (Jensen, 2013). Email requests were sent to organizations with contact details in the 2013 situational analysis.

We also searched locally at Myanmar universities including University of Medicine 1, University of Medicine 2, University of Yangon Department of Psychology and Department of Social Work, University of Mandalay, and the Defense Services Medical Academy to identify gray literature and published articles in both Myanmar and English languages. Literature was also requested from the Myanmar Psychological Association, Myanmar Mental Health Society, and Myanmar Medical Association.

For all searches, search terms included combinations of ‘mental health’, ‘mental illness’, and ‘psychosocial’; for searches in sources not specific to Myanmar, ‘Burma’, ‘Burmese’, and ‘Myanmar’ were also included. The original search was completed in September and October, 2015, and was updated in October and December, 2016.

## Results

### People from Myanmar's perspectives on MH problems

In-depth qualitative studies provide people from Myanmar's perspectives on the nature and experience of MH problems (Cardozo *et al.* 2004; Lim *et al.* 2013; Shannon, 2014; Shannon *et al.* 2014, 2015; Fellmeth *et al.* 2015). Syndrome descriptions illustrate problems similar to those of Western descriptions of stress, anxiety, depression, and mental trauma, but with additional culturally relevant symptoms (e.g. feeling numb, thinking too much, and feeling hot under the skin) (Cardozo *et al.* 2004; Lim *et al.* 2013; Fellmeth *et al.* 2015; Shannon *et al.* 2015). A range of symptom severity has also been described, with thinking too much and keeping thoughts and feelings inside associated with increased severity and risk of suicide (Shannon *et al.* 2015).

People from Myanmar report being unlikely to spontaneously discuss MH problems but open to doing so if asked directly by a respected health professional (Shannon, 2014; Shannon *et al.* 2014). Talk-based counseling services available in refugee camps have been utilized although perspectives on this type of treatment is mixed; some people report improvement whereas others discuss stigma and poor understanding of counseling as barriers to care (Cohen & Asgary, 2016). Concerns about being hospitalized and a reticence to complain about their problems to others may impact willingness to disclose problems (Shannon, 2014; Shannon *et al.* 2014). Self-reflection (often tied to meditation and religion), mindfulness/acceptance, and social and community engagement are discussed as coping strategies (See, 2013; Cohen & Asgary, 2016).

These qualitative findings, although limited, suggest that MH problems experienced by people from Myanmar are similar to Western syndromes that can be effectively treated by psychotherapeutic interventions. The findings of positive views toward health professionals, participating in daily tasks and activities, and counseling speak to the appropriateness of psychotherapy.

### MH services in Myanmar

MH expenditures account for 0.3% of all health expenditures (WHO & Ministry of Health, 2006). Nearly 75% of the MH budget is for mental hospital expenditures (WHO, 2011). MH services are provided primarily through two psychiatric hospitals, 22 psychiatric wards of general hospitals, and 35 outpatient MH facilities (WHO, 2015). Primary care physicians can prescribe psychiatric medications and have access to MH treatment manuals, but the majority have not received training on MH within the past 5 years (WHO, 2011). For every 100 000 people, only 0.6 trained MH workers (e.g. psychiatrists, psychiatric nurses) are available, and only 16% of these work in outpatient settings. For comparison, there are 125.2 per 100 000 in the USA and 318.9 per 100 000 in the UK (WHO, 2015).

The World Health Organization (WHO) advocates for the integration of MH services into primary care and other community-based delivery platforms to improve access and lower cost (Funk & Ivbijaro, 2007; Patel *et al.* 2013). Taken together, data from the WHO resources (WHO & Ministry of Health, 2006; WHO, 2011) indicate a lack of human resources for community-based therapeutic interventions within the formal healthcare system. The historical role of the psychiatric hospitals in Myanmar, paired with the likelihood of prolonged hospitalization if committed, may reduce help-seeking behaviors (Kent, 1996; Way, 1996; Zaw, 1997). The use of informal service networks for MH care is not well documented, although anecdotal reports suggest that people in distress may receive counseling support in monastic settings and meditation centers (Way, 1996). Most of the documented psychosocial and psychotherapeutic interventions have been provided by various non-governmental and community-based organizations, often by lay providers or community health workers (Risso-Gill *et al.* 2014).

### Assessment of MH interventions

We identified evaluations of 14 MHPSS interventions delivered either within Myanmar or to people from Myanmar living outside the country. Table 1 provides an overview of the included studies. The three community-level interventions all tended to be broadly psychosocial and aimed at promoting community wellbeing rather than reducing distress. These programs were the least likely to have been objectively evaluated, resulting in largely anecdotal support for their effectiveness (Paratharayil, 2010; Ma, 2011; Letzelter & Gimenez, 2015). Three reports focused on adult religion-based practice (meditation), two of which included only a comparison with a matched, non-randomized control group; the third not utilizing a control group at all (Kyaing, 2002; Aye, 2007; Kasai *et al.* 2017). There was greater support for and documentation of psychotherapeutic interventions targeting adults with particular risk factors or elevated symptoms. Of the four interventions reviewed (Paratharayil, 2010; van Wyk *et al.* 2012; Bolton *et al.* 2014; Vukovich & Mitchell, 2015), all included Western psychotherapy elements, one was delivered in group format (Vukovich & Mitchell, 2015), three were implemented with lay counselors (Paratharayil, 2010; Bolton *et al.* 2014; Vukovich & Mitchell, 2015), and two included a standardized implementation manual (Bolton *et al.* 2014; Vukovich & Mitchell, 2015). Three of these provided objective assessment scores demonstrating symptom reduction (van Wyk *et al.* 2012; Bolton *et al.* 2014; Vukovich & Mitchell, 2015), though only one included a comparison group and locally validated measures (Bolton *et al.* 2014). Two reports evaluated interventions aimed at symptom reduction in children (Rowe *et al.* 2016). Two other child interventions focused on promoting wellbeing or post-traumatic growth, and did not include pre–post assessments to measure change (Prag & Vogel, 2013; Tanaka, 2013).

**Table 1.** Summary of intervention studies conducted with people from Myanmar

Author (Year)	Study population	Intervention description	Outcomes reported	Key limitations
Annan *et al.* (2016)	479 families from Myanmar living on the Thai–Myanmar border	A locally adapted version of the evidence-based *Strengthening Families Program*: 12 weekly sessions of 2 h each in which parents and children participated separately in skills groups for the first hour, and then came together for supervised practice and interaction for the second hour	Significant reductions in child externalizing problems (reported by both children and adults) and attention problems (reported by adults) among treatment children compared with wait-list control children as measured by the Child Behavior Checklist and Youth Self-Report; significant increase in psychosocial protective factors as reported by children	Comparison group was a wait-list control; as a meal was provided at each session, an active control such as meal participation would ensure the effects are attributable to the intervention itself
Aye (2007)	174 people attending 10-day meditation courses at three different centers	10-day Vipassana meditation training at three different Buddhist meditation centers: approximately 10 h of meditation practice per day with alternating sitting and walking sessions. During sitting sessions, practitioners attempt to concentrate on their breathing or specific objects provided by instructor. Walking meditation focused on moment-to-moment awareness	Significant reductions in anxiety, stress, and aggression and an increase in self-confidence using the Self-Rating Anxiety Scale, Personal Distress Scale, Frustration and Aggression Scale, and the Bernreuter Personality Inventory	No comparison group, differences in intervention instructions between centers
Bolton *et al.* (2014)	347 Burmese Refugees in Thailand with trauma exposure and elevated depression and/or post-traumatic stress	Common Elements Treatment Approach (CETA): a manualized psychotherapy that incorporates components common to many evidence-based treatments. Typically consisting of 8–12 sessions with components selected according to the client's symptom presentation	Significant pre–post reduction in symptoms of depression, post-traumatic stress, anxiety, and functional impairment relative to a wait-list control group. No significant reduction in substance use	Loss to follow-up was higher than in comparable studies elsewhere
Kasai *et al.* (2017)	97 people from Myanmar practicing meditation and 81 Myanmar nurses with no meditation experience	Vipassana meditation at a Buddhist monastery: 1 h sitting meditation sessions occurring 10 times per day. During contemplation practitioners attempt to sit silently with clear minds and concentrate only on their breathing. Participants had been practicing meditation from <1 year to more than 7 years	Participants in the meditation group had significantly lower depression, anger, and fatigue scores, and significantly higher vigor scores, than the comparison nurses using the Profile of Mood States – Short Form. No difference in confusion or tension/anxiety. No overall difference between groups in psychological flexibility measured by the Acceptance and Action Questionnaire II, although scores on this measure decreased (indicating greater psychological flexibility) with increasing length of meditation experience	Group comparisons made based on cross-sectional assessment (no pre–post) among respondents who self-selected into either the meditation or control condition, precluding causal inference
Kyaing (2002)	200 participants in a 10-day meditation course and 200 service personnel at an auto industry enterprise	10-day Vipassana meditation training at a Buddhist meditation center: 10 h of guided sitting meditation per day. Silence is practiced while not meditating. During contemplation practitioners attempt to sit silently concentrating only on their breathing for 3.5 days. This is followed by 6.5 days of attempting to directly observe the changing nature of body and mind	Significant pre–post reductions in anxiety and stress for Vipassana course participants, whereas control group reported increases using the Self-Rating Anxiety Scale and the Personal Distress Scales	Author noted difficulties in finding a matched control group for comparison. Participants self-selected into the meditation condition
Letzelter & Gimenez (2015)	1161 adults from Rakhine State, Myanmar receiving humanitarian support from ACF International	Support and self-help groups to enhance maternal and child care. Participants grouped in three categories: women, men, and mother in-laws. Average of seven sessions conducted per group, with groups meeting once every 2 weeks. Focus of the groups was to find solutions to enhance child, woman, and family wellbeing through care practices and increased support for women at family level	Pre–post assessments were completed using the Self-Reporting Questionnaire (SRQ-20) and qualitative data were analyzed with the Most Significant Change (MSC) technique. Improvements in SRQ-20 scores were reported in all groups: women, men, and mother in-laws	No comparison group, disparity in MSC results between sites attributed to misunderstandings by group facilitators in methodology and collection instructions
Ma (2011)	Community of Burmese immigrants in Australia	Community music band developed through a participatory research approach to address social isolation and internal group fragmentation	Qualitative feedback led to the author's conclusion that the band helped to develop social solidary	No comparison, no formal program evaluation
Paratharayil (2010)	Communities in Myanmar hit by Cyclone Nargis	Community kitchens: provision of nutritious meals and a safe space for collective sharing, facilitated by community leaders	Anecdotal reports of improved psychosocial wellbeing	No pre–post assessment reported
Paratharayil (2010)	Cyclone Nargis survivors in Myanmar who were having difficulty coping	Focused trauma care: up to 12 individual sessions including aspects such as psychological first aid, basic needs assessment, supportive counseling, and transcendental meditation	Anecdotal reports of improved ability to cope with grief	No pre–post assessment reported
Prag & Vogel (2013)	Nine Shan adolescents age 16–19 living in a refugee camp in Thailand	Therapeutic photography: a 5-week photojournalism class in which assignments encouraged students to examine their own stories, with weekly group discussions using therapeutic conversation techniques	Qualitative themes of post-traumatic growth found to be similar to themes found elsewhere, but with the addition of a cluster of responses related to an ‘ability to articulate the social narrative’ that was important to the students	No pre–post assessment to assess whether post-traumatic growth occurred, no comparison group
Rowe *et al.* (2016)	30 school-aged Burmese refugees in the USA (age 11–20)	Burma Art Therapy Program (BAPT): 16 individual art therapy sessions of 50 min each delivered over a 6-month period. Sessions were a mix of structured and unstructured	Significant pre–post reduction in median anxiety scores using the Hopkins Symptoms Checklist (magnitude of change not reported). Change in depression scores not reported. Minor improvements in self-concept indicators (Piers-Harris Self-Concept Scale) and minor decrease in teacher-reported student difficulties (Strengths and Difficulties Questionnaire) were not statistically significant. Provider focus groups reported improved child wellbeing that they felt were not captured in quantitative assessments	No comparison group, small sample size, not all comparisons reported
Tanaka (2013)	Karen children aged 2–5 attending nursery schools in Karen refugee camps in Thailand	Nursery school program designed to promote wellbeing through a child-centered curriculum in which teachers provided emotional and social support, promoted socialization and cooperation, and used positive discipline	Longer attendance predicted higher scores on positive development but was not associated with emotional/behavioral problems	Assessment conducted at a single time point; assessing impact by comparing children by duration of attendance is likely to be confounded by other factors
Van Wyk *et al.* (2012)	62 newly arrived Burmese refugees in Australia	Individualized resettlement program consisting of assessment, social assistance, referrals, and possible exposure to a variety of therapeutic interventions (including psychoeducation and skills-based, expressive, supportive, couples/family, cognitive behavioral, and exposure therapies)	Significant pre–post reductions in post-traumatic stress disorder, anxiety, and depression assessed using the Harvard Trauma Questionnaire, Hopkins Symptom Checklist, and Post-Migration Living Difficulties checklist	No comparison group, guidelines for intervention decisions not clearly outlined
Vukovich & Mitchell (2015)	57 PLWHA, LGBTQ, and former political prisoners living in Myanmar	Sharing Circles: a manualized eight-session psychotherapy group consisting of interactive and narrative tasks, body mapping, role playing, gestalt psychotherapeutic activities, lifeline and memory book, meditation, and self-care	Significant pre–post reductions in depression symptoms and psychosocial stressors assessed using the Brief Patient Health Questionnaire	No comparison group

#### Community-level psychosocial interventions

The first community-level intervention was a community kitchen program delivered in areas of Myanmar affected by cyclone Nargis, which brought people together for nutritious meals while providing a safe space for collective sharing, story-telling, and grieving (Paratharayil, 2010). The second program was a community band developed through a participatory research approach among resettled refugees in Australia (Ma, 2011). Both programs were reported to contribute to community wellbeing, although no formal program evaluations were attempted.

A third program established support and self-help groups to enhance maternal and child care in complement to a larger nutrition program (Letzelter & Gimenez, 2015). The groups were divided into three categories to promote communication – women, men, and mother in-laws – with 1161 participants. An average of seven sessions were conducted per group, with groups meeting once every 2 weeks. The focus of the groups was to enhance child, woman, and family wellbeing through care practices and increased support for women. Pre- and post-assessments were completed using the Self-Reporting Questionnaire. Improvements in scores were reported in all groups: women (5.1 *v.* 2.0), men (5.4 *v.* 3.5), and mother in-laws (5.2 *v.* 2.9), though statistical significance was not presented and the study lacked a comparison group.

#### Adult religion-based practice

Kasai *et al.* (2017) conducted a cross-sectional survey comparing 97 practitioners of Vipassana contemplation to a matched control group of 81 hospital nurses who had no previous meditation experience. Those in the meditation group practiced sitting meditation for roughly 10 h per day. The survey assessed a number of mostly negative moods using the Profile of Mood States – Short Version. Compared with non-meditating nurses, Vipassana practitioners reported less depression and dejection (5.1 *v.* 7.0, *p* = 0.002), anger and hostility (5.0 *v.* 7.9, *p* < 0.001), and fatigue (5.4 *v.* 7.9, *p* < 0.001), and more vigor (14.2 *v.* 12.4, *p* < 0.001). There were no significant differences between the groups in tension and anxiety, confusion, or psychological flexibility. While supported by anecdotal accounts of the benefits of meditation, this study is limited by using only a cross-sectional comparison of two naturally self-selecting groups, thereby lacking the ability to document change or attribute it to the intervention.

Two additional studies focusing on the effects of Vipassana meditation on anxiety and stress were conducted as theses for Myanmar postgraduate students. In each study, the population consisted of participants in 10-day Vipassana meditation courses. Two scales – the Self-Rating Anxiety Scale and the Personal Distress Scale – were common to both studies. Myint Myint Aye also used the Frustration and Aggression Scale from the Mandalay Personality Index and a Myanmar adaptation of the Bernreuter Personality Inventory to measure sociability and self-confidence.

Kyaing (2002) compared 200 participants of a 10-day Vipassana meditation course with a matched control group of 200 service personnel from a government automobile enterprise. The meditation group practiced sitting meditation for approximately 10 h per day. Following the intervention, participants in the Vipassana course reported reductions in anxiety (14.16 *v.* 7.75, *p* < 0.001) and stress (33.05 *v.* 19.43, *p* < 0.001), whereas the control group reported increases in both anxiety (9.135 *v.* 10.24, *p* < 0.001) and stress (24.93 *v.* 27.55, *p* < 0.001) during the same 10-day period. Participants in the Vipassana course were in an environment constructed to minimize external stressors whereas the control group remained in their daily work environment, limiting our ability to attribute change to the meditation *v.* other aspects of monastery life.

In a separate study, Aye (2007) conducted pre–post interviews with 174 participants in 10-day Vipassana meditation courses at three different meditation centers. Participants again reported reductions in anxiety (8.12 *v.* 12.54, *p* < 0.05), stress (12.52 *v.* 21.91, *p* < 0.01), and aggression (6.95 *v.* 8.58, *p* < 0.01), and an increase in self-confidence (7.86 *v.* 7.06, *p* < 0.01) following the course. No significant changes were reported for frustration and sociability. This study faced the same limitations as the prior two and did not include a control group.

#### Adult psychotherapeutic interventions

The first adult program focused on providing psychosocial support and trauma care to survivors of cyclone Nargis who were showing symptoms of distress (Paratharayil, 2010). Local ‘trauma care givers’ were trained to deliver up to 12 individual counseling sessions including aspects of psychological first aid, basic needs assessment, listening skills, group work, and transcendental meditation. The Impact of Event Scale was used to screen individuals and guide the development of a treatment plan. Anecdotal reports of positive outcomes included improved understanding of trauma reactions and ability to cope with grief; however, no follow-up assessment was reported, there was no comparison group, and the scale used may not have been validated in the local culture (Paratharayil, 2010).

Vukovich & Mitchell (2015) reported outcomes of the Sharing Circles program, focused on improving psychological symptoms and providing psychosocial support to members of vulnerable groups in Myanmar. The eight-session program involved monthly group meetings with a topic and activity for each session, including: (1) *engagement and establishing safety*; (2) *life narrative*; (3) *discovering our identity*; (4) *communicating with support systems*; (5) *river of life activity*; (6) *our dignity, our value*; (7) *future dreams*; and (8) *closing ceremony*. The study included 57 men and women who were either (1) living with HIV/AIDS, (2) identifying as LGBTQ, or (3) former political prisoners. Pre–post assessments were conducted using the Brief Patient Health Questionnaire. Following the intervention, the group experienced small but statistically significant reductions in depression symptoms (14.26 *v.* 15.79, *p* = 0.01) and psychosocial stressors (12.74 *v.* 14.46, *p* = 0.035). Future evaluation of this program would benefit from the addition of a comparison group.

The two interventions described above were both implemented within Myanmar. Van Wyk *et al.* (2012) conducted a naturalistic study evaluating the MH impact of a refugee resettlement program in Australia. In this study, 62 newly arrived Burmese refugees received a standard assessment and were subsequently provided social assistance, referrals, and therapeutic interventions (e.g. psychoeducation, skill-based therapies) according to the typical program practice. Participants were assessed at pre- and post-intervention periods regardless of their level of engagement using the Harvard Trauma Questionnaire, Hopkins Symptom Checklist, and Post-Migration Living Difficulties checklist. Pre–post assessments showed significant decreases in the proportion of participants experiencing post-traumatic stress disorder (5% *v.* 27%), anxiety (5% *v.* 23%), and depression (7% *v.* 37%). However, changes in these scores were not related to the number of service contacts and there was no comparison group, making it difficult to separate the impact of the interventions from potentially confounding factors.

Lastly, the Common Elements Treatment Approach (CETA) is an 8–12 session intervention administered by lay workers that incorporates elements that are common across many evidence-based treatments (Murray *et al.* 2014). Trauma-affected people from Myanmar who met severity criteria for depression and/or PTS were randomly assigned to receive treatment (*n* = 182) or to a wait-list control condition (*n* = 165) (Bolton *et al.* 2014). MH symptoms, substance use, and functional impairment were evaluated using a locally validated assessment tool (Haroz *et al.* 2014). Compared with the wait-list group, those receiving CETA experienced reductions of 77% in depression, 76% in anxiety, 75% in PTS, 67% in functional impairment, and 71% in aggression (*v.* reductions of 40, 41, 37, 22, and 32% for respective outcomes in the control group). There was no significant difference in change scores for substance use. This evaluation provides greater evidence of effectiveness due to the inclusion of a control group to help isolate the impact of treatment to other potential factors; however, with no active comparison, the impact of particular intervention components cannot be separated from other features of supportive counseling.

#### Child-focused interventions

Two child-focused interventions targeted symptom reduction. Annan *et al.* (2016) reported on a randomized controlled trial of an adapted evidence-based parenting intervention (Kumpfer *et al.* 2005). Study participants included 479 families from Myanmar living on the Thai–Myanmar border; families were randomly assigned to either the intervention or a wait-list control group. The adapted intervention consisted of 12 weekly sessions of 2 h each in which caregivers and children participated separately in parenting and social skills groups for the first hour, and then came together for a second hour of interactive skills practice and feedback. Program adaptations were based on prior qualitative work and the intervention was delivered by locally recruited lay facilitators. Outcome measures included the Achenbach Child Behavior Checklist and Youth Self-Report and a locally developed assessment of child psychosocial protective factors. Pre–post assessments showed small but significant improvements in externalizing problems, attention problems, and psychosocial protective factors among intervention children as compared with control children; no significant improvements were reported for internalizing problems. The authors highlighted the feasibility and acceptability of this parenting intervention in the target population, but also noted the intensive training, monitoring, and supervision required to support intervention adaptation and fidelity issues.

Rowe *et al.* (2016) reported outcomes of the Burma Art Therapy Project, in which 30 refugees (age 11–20 years) from Myanmar living in the USA received an average of 16 sessions of art therapy, either individually or in groups, in schools over a 6-month period. Youth were assessed before and after the intervention using the Strengths and Difficulties Questionnaire, Hopkins Symptom Checklist, Harvard Trauma Questionnaire, and Piers–Harris Self-Concept Scale At baseline, 40% of participants met criteria for depression and 20% for anxiety. The authors report a significant decrease in median anxiety scores (*p* < 0.0001) but the magnitude of the change is not reported. Changes in depression scores, positive self-concept, and severe difficulties were not statistically significant. The authors discuss the possibility of cultural differences impacting measurement and note that providers felt the deficit-based assessment instruments failed to capture the positive growth that occurred during treatment. This discrepancy highlights the importance of clearly defining and objectively assessing performance indicators that reflect treatment goals.

Two additional child-focused programs were designed to promote positive health and development. Tanaka (2013) reported a cross-sectional evaluation of a nursery school program for 350 young children that aimed to promote wellbeing. Teachers rated children using a locally developed instrument assessing both positive psychosocial development and emotional/behavioral problems. Longer attendance predicted higher average scores on the positive development scale but was not associated with the problem scale. While this may be suggestive that the nursery school was effective in promoting psychosocial development, lack of pre–post assessments or a control group precludes quantifying change.

A second child MH promotion intervention was a 5-week therapeutic photography program on post-traumatic growth among Shan adolescent refugees in Thailand (Prag & Vogel, 2013). The program involved nine adolescents participating in a photojournalism class which included assignments and therapeutic conversation techniques to help students examine their own stories. One year after the end of the program, a clinical psychologist assessed six available participants and analyzed their qualitative responses for themes related to post-traumatic growth. A cluster of responses related to an ‘ability to articulate the social narrative’ (p. 41) was reported to be important to the students. This study was not designed to measure change or attribute outcomes to the intervention.

### Perspectives of Myanmar MH providers

A psychiatrist of Myanmar origin has linked the strong Buddhist tradition in Myanmar to the appropriateness of psychotherapy, explaining that Buddhist teaching identifies the mind as the source of happiness and misery (Way, 1996). The influence of Buddhist practice is reflected in the interest of graduate students in Myanmar on the potential MH benefits of meditation (Kyaing, 2002; Aye, 2007; Kasai *et al.* 2017).

Provider perspectives on psychotherapeutic interventions have generally been positive (James, 2012; Lee, 2012; Win, 2014). Provider observations on the benefits of community engagement activities and client monitoring data, and the heavy burden of stressors that providers themselves face, suggest that program aspects such as community outreach, monitoring and evaluation (M&E), clinical supervision, and self-care should be incorporated into program planning.

## Discussion

Both qualitative and quantitative studies suggest that people from Myanmar experience MH problems due to stress that are similar to those in other populations for whom psychotherapeutic interventions are shown to be effective. While this supports the appropriateness of similar services for people from Myanmar, very little information has been published about the availability or effectiveness of psychosocial and psychotherapeutic interventions in Myanmar. Much of what *is* available consists only of preliminary evaluations of programs delivered by non-governmental or community-based organizations, with few details on the specific activities of the program. While these programs are said to be well received, anecdotal reports of suitability and effectiveness are rarely coupled with rigorous outcome evaluations.

At best, most of the programs reviewed here reported a pre- and post-assessment to document symptom changes. Changing circumstances during implementation and the lack of a control or comparison group leaves us unable to draw strong conclusions about program effectiveness. Counterfactual studies are needed. Evaluation of future programs would be strengthened by including a clear M&E plan from the beginning of program development. Manuals and guidebooks are available to help programs develop an M&E plan (see, e.g. United Nations Development Programme, 2009; International Federation of Red Cross and Red Crescent Societies, 2011). Additional guidance on M&E specific to MHPSS programs is also available (see, e.g. Department of Mental Health-Johns Hopkins School of Public Health and World Bank, 2015; Applied Mental Health Research Group (AMHR), 2013). To facilitate knowledge sharing, program reports should include detailed information about intervention content and implementation.

While most programs that included assessments reported use of appropriate translation methods and efforts to ensure relevance of the items, many either did not discuss whether the instruments were locally validated (e.g. Paratharayil, 2010; Kasai *et al.* 2017) or acknowledged that the use of unvalidated measures made it difficult to interpret or draw conclusions from their results (e.g. Annan *et al.* 2017, Rowe *et al.* 2016). Even commonly used measures should be validated for use in a new population for whom symptom presentations and idioms of distress may differ (Bass *et al.* 2007). Balancing the need for sound assessment measures against programmatic demands can be challenging, but lack of valid measures limits ability to objectively demonstrate change. The Design, Implementation, Monitoring, and Evaluation (DIME) manual provides guidance on validating instruments in challenging settings (Applied Mental Health Research Group, 2013) The WHO toolkit for assessing MHPSS needs in humanitarian settings is another helpful resource (WHO & UNHCR, 2012).

We found less research assessing child MH needs or evaluating therapeutic approaches for children and youth from Myanmar. There is a need for valid methods of identifying children and adolescents in need of therapeutic intervention. A 2013 mapping exercise includes a compendium of tools and approaches for assessing child MH and psychosocial wellbeing (Ager *et al.* 2014) that could be adapted and validated for use in Myanmar. Programs aimed at promoting child wellbeing would also be strengthened by more clearly identifying appropriate objectives and outcome measures. The family intervention reported by Annan *et al.* (2017) aimed to address both symptom reduction and promotion of wellbeing; although the effect sizes from that intervention were small, the adaptation and evaluation process is one that should inform future efforts.

This review is based on a rigorous search of peer-reviewed and gray literature. Future research should include interviews with stakeholders to provide additional insight into the role of culture on MH, the state of MH resources, and the nature of MH professional practice in Myanmar.

## Conclusion

The goal of this review was to summarize the available written information on the suitability, availability, and evidence for MHPSS programming in Myanmar in order to advance research and development in this area and to improve existing and future services. We found very few intervention evaluations, most of which were of limited value in evaluating effectiveness due to methodological constraints. Additional research is needed to assess the diverse and locally untested forms of MHPSS programming offered in Myanmar. Most adult MH programs that have been evaluated in other countries are based on cognitive–behavioral principles and have some support for their efficacy, but local effectiveness is yet to be demonstrated in Myanmar. Other forms of individual coping, such as the use of meditation, have anecdotal support and may be useful in overcoming common service barriers, but more rigorous research is needed to establish the effectiveness of these types of supports in reducing mental distress. Evaluation of programs to reduce child mental distress is particularly lacking and should be prioritized. Incorporating additional research design elements into future program evaluations could help build evidence for intervention effectiveness.
